# Excessive bowel volume loss during anus-preserving surgery for rectal cancer affects the bowel function after operation: A prospective observational cohort study (Bas-1611)

**DOI:** 10.1016/j.heliyon.2023.e17630

**Published:** 2023-07-06

**Authors:** Fan Liu, Peng Guo, Quan Wang, Fujun Chen, Wenyong Wu, Xiangqian Su, Guiying Wang, Zhouman Yu, Jianlong Jiang, Feng Liang, Dechang Diao, Zhikang Chen, Yuanting Liu, Fanqiang Meng, Ning Ning, Yingjiang Ye

**Affiliations:** a Department of Gastroenterological Surgery, Peking University People's Hospital, Beijing, China; b Department of Gastrointestinal Surgery, The First Hospital of Jilin University, Changchun, China; c Department of Colorectal Surgery, The First Affiliated Hospital of Jiamusi Medical University, Jiamusi, China; d Department of General Surgery, Anhui No. 2 Provincial People's Hospital, Hefei, China; e Department of Gastrointestinal Surgery IV, Peking University Cancer Hospital, Beijing, China; f Key Laboratory of Carcinogenesis and Translational Research, Peking University Cancer Hospital, Beijing, China; g Department of General Surgery, 3rd Hospital of Hebei Medical University, Shijiazhuang, China; h 2nd Department of General Surgery, 4th Hospital of Hebei Medical University, Shijiazhuang, China; i Department of Gastroenterological Surgery, Qilu Hospital of Shandong University (Qingdao), Qingdao, China; j Department of General Surgery, Changshu Hospital Affiliated to Soochow University, First People's Hospital of Changshu City, Changshu, China; k Department of General Surgery, The Fifth Medical Center of Chinese PLA General Hospital, Beijing, China; l Department of Gastrointestinal (Tumor) Surgery, Guangdong Province Hospital of Chinese Medicine, The Second Affiliated Hospital of Guangzhou University of Chinese Medicine, Guangzhou, China; m Department of Colorectal and Anal Surgery, Xiangya Hospital of Central South University, Changsha, China; n Department of Gastroenterological Surgery, Tangshan People's Hospital, Tangshan, China; o Department of General Surgery, China-Japan Friendship Hospital, Beijing, China; p Department of Gastrointestinal Surgery, Peking University International Hospital, Beijing, China

**Keywords:** Rectal cancer, Postoperative bowel function, Low anterior resection syndrome, Bowel volume loss, Surgical margin

## Abstract

**Background:**

Bowel volume loss during anus-preserving surgery (APS) may result in low anterior resection syndrome (LARS). We conducted this prospective observational cohort study to measure the incidence of LARS after surgery and evaluate the relationship between bowel volume loss and bowel function.

**Methods:**

Patients with R0 resectable rectal cancer who consented to several bowel function surveys through telephone interviews after the operation were included. Enrolled patients underwent standard APS for rectal cancer, and three length indexes, viz. length of excised bowel, length of the distal margin and length of the proximal margin (LPM) of fresh bowel specimens, were measured in vitro.

**Results:**

The three measured variables of the specimens showed a positively skewed distribution. Patient interviews revealed a trend of gradual improvement in bowel function. Univariate analyses revealed that longer LPM was associated with a significantly negative impact on bowel function at all time points. In multivariate analysis, LPM was found to be a significant risk factorstatistically significant, but its impact was not as strong as that of radiotherapy and low-middle tumour. Furthermore, there was no significant difference in the lymph node detection rate between <10-cm and ≥10-cm LPM groups.

**Conclusion:**

In APS for rectal cancer, bowel volume loss is an important factor causing postoperative bowel dysfunction. Controlling LPM to <10 cm may help improve postoperative bowel function.

## Highlights

• The first cohort to clarify the negative effect of bowel volume loss on post-operative bowel function for rectal cancer. The length of the proximal margin (LPM) serves as a crucial indicator of bowel volume loss.
• The shorter LPM is associated with better postoperative bowel function.
• The surgeon can enhance postoperative bowel function proactively by reducing the LPM.

## 1 Introduction

In China, approximately 50% of diagnosed colorectal cancers are located in the patient's rectum [1]. Advanced surgical techniques and comprehensive treatment strategies have helped several patients with rectal cancer undergoing anus-preserving surgery (APS) achieve long-term survival after surgery. Low anterior resection syndrome (LARS) is a common functional disorder that develops in patients with rectal cancer after they undergo APS [2,3]. Its symptoms include changes in bowel movement frequency, rhythm disorder, incontinence and constipation, which have been confirmed to seriously affect patients' postoperative quality of life [4]. Approximately 30%–55% of patients with rectal cancer have severe LARS symptoms after complete APS, which can last for several years [5,6].

Radical resection consistent with total mesorectal excision (TME) standards requires the removal of a part of or the entire rectum and sigmoid colon, leading to inevitable bowel volume loss, which may result in LARS symptoms. Previous studies have reported that anorectal manometry can be used to confirm the reduction of neorectal volume in patients with major LARS [[7], [8], [9]]; however, the specific underlying mechanism remains unclear. According to this phenomenon, since the 1980s, improved anastomosis methods such as J-pouch anastomosis and transverse coloplasty have been developed to amplify the stool storage capacity of the neorectum [10,11]. However, Marti et al. and Parc et al. suggested that the effectiveness of these remedies is uncertain and that they sometimes even result in new problems, such as emptying dysfunction [12,13]. Thus, merely increasing the neorectal volume is not sufficient because most of the moulding stool is stored in the sigmoid colon, where it is difficult to restore the lost volume using existing surgical techniques [14].

Another flaw in this clinical practice is that current clinical techniques are not yet capable of quantifying bowel volume loss [15]. Direct measurement of postoperative bowel volume is difficult and uncertain because of the extensibility of the bowel wall. The probe length of anorectal manometry is generally short; hence, it is difficult to evaluate the residual sigmoid colon. Shafik et al. placed long probes in volunteers’ colons through colonoscopies; however, this approach is cumbersome and requires anaesthesia, making it difficult for clinical use [16]. Existing evidence indicates that the methods of placing a balloon or manometry probe to measure capacity are defective [17].

Removal of a longer bowel certainly results in more bowel volume loss. Measuring the length of the bowel specimens removed during surgery can directly reflect bowel volume loss, and the length is also comparable among different individuals. Based on this theoretical hypothesis, we designed a prospective observational cohort study (BaS-1611) to measure the anatomical length of the bowel removed during APS using a standardised method, observe the incidence of LARS after surgery and evaluate the relationship between bowel volume loss and bowel function.

## 2 Methods

### 2.1 Study population

BaS-1611 is a multi-centre, prospective, observational cohort study involving 14 Chinese medical centres, including 8 affiliated university hospitals, 4 regional central hospitals, 1 military hospital and 1 provincial hospital of traditional Chinese medicine. Patients with histologically confirmed rectal adenocarcinoma (0–12 cm from the anal verge as confirmed by rigid sigmoidoscopy), aged ≥18 years and awaiting R0 resection and primary reconstruction were prospectively enrolled in this study from January 2017 to April 2018. Patients with emergency situations, cognitive impairment, non-primary anastomosis, previous history of left colon or anorectal surgery and long-term preoperative bowel dysfunction were excluded. Exclusion criteria also included anastomosis recurrence during follow-up, death before the first follow-up and permanent stoma during follow-up.

### 2.2 Registration

BaS-1611 has been approved by the ethics committee of the competent authority (2017PHB011-01) and registered on the ClinicalTrial.org website (NCT03009747). All enrolled patients provided complete signed informed consent. For real-time patient registration and management of research data, the researchers used a rented and customised project management network platform (https://www.boshicloud.com) that was shared by all the research centres.

### 2.3 Study design

The enrolled patients underwent standard APS for rectal cancer and were followed up through three telephone interviews at 3 (90 days), 6 (180 days) and 12 months (365 days) after surgery. The study did not recommend the length or site of bowel resection, and surgeons were encouraged to make free decisions based on TME principles and surgical safety. The follow-up included patients’ survival status, cancer treatment programme, subjective bowel function survey and LARS scores [18].

### 2.4 Specimen measurements

Fresh bowel specimens were measured within 30 min in vitro. Surgeons were asked to dissect the bowel lengthwise and tensionless flatten it, place a ruler next to the specimen and then take photographs and upload them to the platform. These photographs were evaluated independently by two trained surveyors according to the same standard (Appendix 1), and results were averaged and recorded on the platform. The measurement included three length indexes, viz. length of excised bowel (LEB), length of the distal margin (LDM) and length of the proximal margin (LPM). Measurements were performed using the Digimizer software (version 4.2.6.0, MedCalc Software, Belgium).

### 2.5 Bowel function interview

All patients were interviewed by a third-party follow-up team that was employed and trained in functional follow-up. The interview was appropriately audio-recorded and stored offline to ensure the traceability and quality of the research data. The recorded data were rechecked by experts of the research group against the interview results before the data were statistically analysed to guarantee the accuracy of the telephone interview.

Owing to the particularity of conducting interviews by telephone, we formulated rules to ensure the authenticity, objectivity and traceability of the interview data. For instance, the follow-up investigator must follow the order of questions in the questionnaire and should not answer on behalf of patients based on their descriptions. All telephone interviews must be answered by the patient. If a call does not connect, the investigator should change the date (generally the next week) and call again. Three unconnected calls would be deemed lost to follow-up. The novel follow-up method designed by the institute has been published partially [19].

The subjective questionnaire contains three questions addressing the primary complaints, bowel function satisfaction and quality of life restriction, which require a ‘yes’ or ‘no’ answer. The LARS score is a total score questionnaire containing five single-choice questions with a corresponding score for each option. Based on the total score (range 0–42 points) of each patient, the bowel function was categorised from best to worst as follows: no LARS (0–20), minor LARS (21–29) and major LARS (30–42) [20]. The LARS score is characterised by numerical exclusivity among the options; thus, the investigators were required to explicitly ask the participants to choose between different numerical ranges.

### 2.6 Statistical analysis

Length measurements were accurate to 1.0 cm. The Kolmogorov–Smirnov test was used to analyse the distribution characteristics of continuous variables. Normally distributed variables were analysed using *t*-test and ANOVA, whereas non-normally distributed variables were analysed using the Mann–Whitney *U* test and Kruskal–Wallis test. The key indicators obtained using univariate analysis and other recognised risk factors related to LARS (such as radiotherapy, tumour location, body mass index [BMI] and stoma [2,[21], [22], [23], [24], [25], [26]]) were subjected to generalised estimating equation. A p-value of <0.05 was considered statistically significant. All analyses were performed using SAS 9.4 (SAS Institute Inc., Cary, NC).

## 3 Results

A total of 459 patients were recruited in 14 research centres from January 2017 to April 2018. Although 52 patients could not be contacted for a follow-up, data obtained from 376 patients having been approached for at least one follow-up and who had complete clinical information were included in the analysis. Of these 376 patients, 257, 275 and 311 patients were contacted for follow-up at 3, 6 and 12 months, respectively (Fig. 1). Table 1 presents the demographic characteristics of the patients (median age: 62 [range: 26–85] years; men = 217, women = 159; median BMI = 23.0 [range: 15.6–33.3] kg/m^2^. In particular, 13.6% of patients received preoperative radiotherapy, 73.4% had clinical stage ≥ III, and 75.3% did not report limitations in daily activities (ECOG score = 0). The median distance from the lower margin of the tumour to the anal verge was 8.0 (range 1.0–12.0) cm, and the proportion of patients with middle-low rectal cancer (≤10 cm) was 85.4%. The TNM stage was mainly II (27.1%) and III (39.4%), although the clinical TNM stage of 28 patients in the preoperative stage could not be accurately predicted. Furthermore, 51 patients received neoadjuvant radiotherapy, of whom 45 received standard long-course radiotherapy with a planned dose of 45–50 Gy. The remaining five patients received short-course radiotherapy (5 Gy by five doses), and one patient dropped out halfway owing to intolerance (six times radiotherapy in total). Another 24 patients also received neoadjuvant chemotherapy, and 10 patients received only preoperative chemotherapy.

**Fig. 1:**
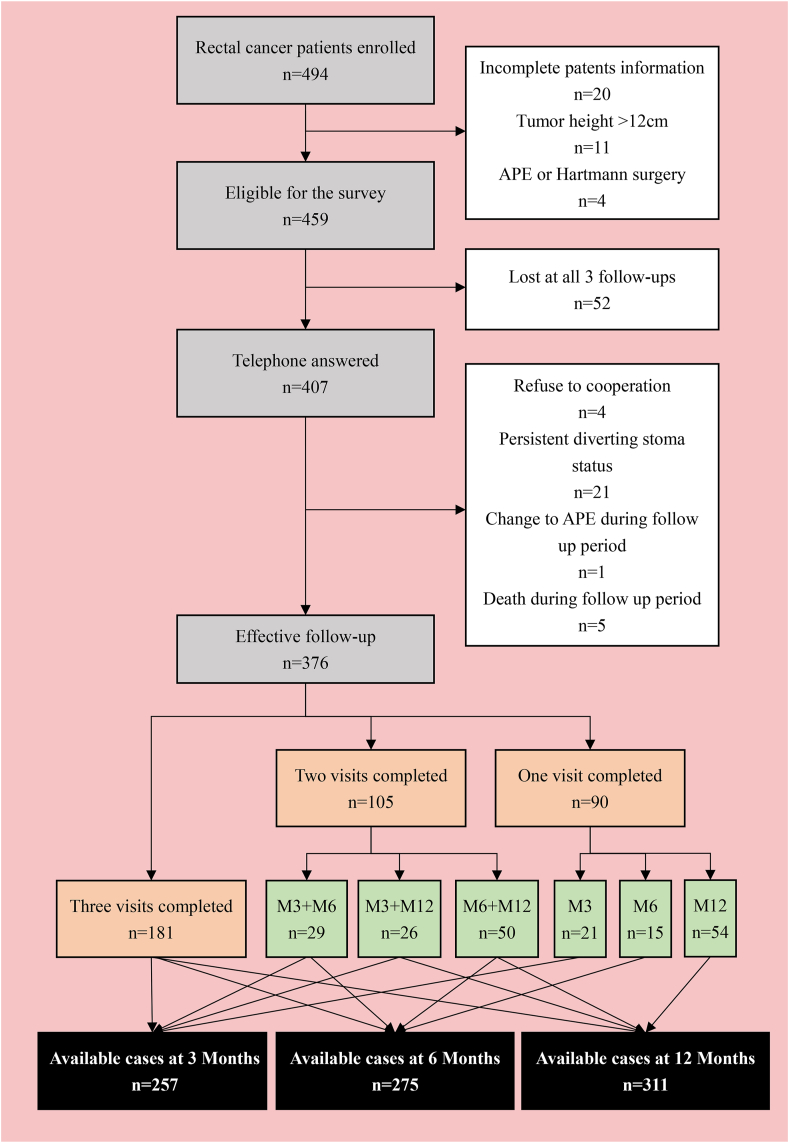
Flow chart depicting the follow-up of 376 patients.

**Table 1:** Demographic data of rectal cancer patients in BaS-1611.

Variables	
Male, n (%)	217 (57.7%)
Female, n (%)	159 (42.3%)
Age at time of surgery, means ± SD	61.5 ± 10.1 years
BMI, means ± SD	23.3 ± 3.2
Distance to anal verge, median (range)	8.0 (1.5–12.0) cm
Clinical T classification, n (%)	
T0	17 (4.5%)
T1	33 (8.8%)
T2	52 (13.8%)
T3	177 (47.1%)
T4	83 (22.1%)
Tx*	14 (3.7%)
Clinical N classification, n (%)	
N0	188 (50.0%)
N1	91 (24.2%)
N2	67 (17.8%)
Nx*	30 (8.0%)
Clinical M classification, n (%)	
M0	346 (92.0%)
M1	16 (4.3%)
Mx*	14 (3.7%)
Preoperative TNM classification, n (%)	
0	17 (4.5%)
I	65 (17.3%)
II	102 (27.1%)
III	148 (39.4%)
IV	16 (4.3%)
Unable to get a TNM stage^#^	28 (7.4%)
Neoadjuvant radiotherapy, n (%)	
Yes	51 (13.6%)
No	325 (86.4%)
Neoadjuvant chemotherapy, n (%)	
Yes	34 (9.0%)
No	342 (91.0%)
Total lymph node dissection, n (%)	
<12	100 (26.6%)
≥12	273 (72.6%)
Missing	3 (0.8%)

* Preoperative TNM data were missing as these patients did not undergo standard preoperative imaging exams; #Owing to missing data, the TNM stage could not be assessed in 14 patients; BMI: Body mass index

Of the 376 patients, 73 (19.4%) underwent open surgery, while the remaining 303 (80.6%) underwent laparoscopic surgery. End-to-end anastomosis was performed in 336 patients (89.4%), while side-to-end anastomosis was performed in 40 patients (10.6%). Except for one patient who underwent manual anastomosis, all the other patients underwent bowel reconstruction via tube stapler. A total of 86 patients (22.9%) underwent prophylactic loop ileostomy, while 4 patients (1.1%) underwent prophylactic loop colostomy.

The three measured variables of the specimens— LEB, LDM and LPM—showed a positively skewed distribution, with median values of 15.0 (range 6.0–39.0) cm, 3.0 (range 1.0–10.0) cm and 9.5 (range 2.0–32.0) cm, respectively. Interestingly, in the three measurements, values of 5, 10 and 15 cm were reported more frequently than the adjacent values, which may be due to the tendency of the surgeon to round up numbers (Appendix 2).

### 3.1 Bowel function results

#### 3.1.1 Univariate analysis

The positive rates for all three subjective questions at 12 months were lower than those at 3 and 6 months; however, only the quality of life restriction differed significantly (p < 0.001). The median values of LEB and LPM were higher in the positive groups at 6 and 12 months, and the median value of LPM differed significantly in all questions, except for the results of bowel function satisfaction at the 12-month follow-up (Appendix 3).

Similarly, the results of the LARS score exhibited a gradual improvement trend; the proportion of patients with no LARS increased from 47.5% to 61.4%, whereas that of patients with major LARS decreased from 32.7% to 22.5% (p < 0.001). LPM and LEB exhibited similar distribution characteristics in the three follow-up nodes, i.e. the two values in the major LARS group were larger than those in the other two groups, particularly LPM (Table 2). Considering the close relationship between LPM and bowel function, we divided LPM into the following four groups according to length: 0–5, 5–10, 10–15 and > 15 cm. The same distribution pattern was found in all three time nodes, i.e. as the LPM increased, the proportion of patients with major LARS increased while that of patients with no LARS decreased. The difference was statistically significant at 6 months (p < 0.001) and 12 months (p = 0.001; Fig. 2).

**Table 2:** Univariate risk factor analysis for LARS score.

Variables	3 months, n = 257				6 months, n = 275				12 months, n = 311			
	No LARS n = 122	Miner LARS n = 51	Major LARS n = 84	p-value*	No LARS n = 144	Miner LARS n = 41	Major LARS n = 90	p-value*	No LARS n = 191	Miner LARS n = 50	Major LARS n = 70	p-value*
LDM (cm)	3.0 (1.0–10.0)	3.0 (2.0–7.0)	3.0 (1.0–8.0)	0.353^#^	3.0 (1.0–8.0)	3.0 (2.0–8.0)	3.0 (2.0–10.0)	0.465^#^	3.0 (1.0–10.0)	3.0 (2.0–9.0)	2.5 (1.0–8.0)	0.151^#^
LPM (cm)	8.0 (2.0–32.0)	8.0 (2.0–18.0)	10.0 (3.0–25.0)	0.013^#^	7.5 (2.0–27.0)	9.0 (2.0–21.0)	10.0 (3.0–32.0)	<0.001^#^	8.0 (2.0–23.0)	10.0 (3.0–21.0)	10.0 (4.0–32.0)	0.001^#^
LEB (cm)	15.0 (8.0–37.0)	14.0 (7.0–25.0)	16.0 (6.0–32.0)	0.082^#^	15.0 (6.0–39.0)	15.0 (8.0–32.0)	16.0 (8.0–37.0)	0.029^#^	15.0 (8.0–27.0)	15.0 (6.0–27.0)	16.0 (8.0–37.0)	0.035^#^
Diverting stoma												
No stoma	103 (84.4%)	49 (96.1%)	80 (95.2%)	0.006	129 (89.6%)	37 (88.1%)	61 (67.8%)	<0.001	157 (81.8%)	33 (66.0%)	38 (54.3%)	<0.001
Diverting stoma	19 (15.6%)	2 (3.9%)	4 (4.8%)		15 (10.4%)	5 (11.9%)	28 (31.1%)		33 (17.2%)	17 (34.0%)	30 (42.9%)	
Radiotherapy												
Yes	5 (4.1%)	2 (3.9%)	5 (6.0%)	0.566	9 (6.3%)	1 (2.4%)	17 (18.9%)	0.004	17 (8.9%)	7 (14.0%)	22 (31.4%)	<0.001
No	117 (95.9%)	49 (96.1%)	79 (94.0%)		135 (93.8%)	41 (97.6%)	73 (81.1%)		175 (91.7%)	43 (86.0%)	48 (68.6%)	
Tumour position												
≤8 cm	53 (43.4%)	29 (56.9%)	45 (53.6%)	0.068	68 (47.2%)	26 (61.9%)	58 (64.4%)	0.005	92 (47.9%)	32 (64.0%)	55 (78.6%)	<0.001
>8 cm	69 (56.6%)	22 (43.1%)	39 (46.4%)		76 (52.8%)	16 (38.1%)	90 (35.6%)		100 (52.1%)	18 (36.0%)	15 (21.4%)	
BMI	23.0 ± 2.9	22.7 ± 2.7	23.6 ± 3.1	0.154^¤^	22.7 ± 3.2	22.4 ± 2,7	23.7 ± 2,8	0.005^¤^	22.8 ± 3.1	23.6 ± 3.2	24.1 ± 2.9	0.008^¤^

*p-values resulting from Mann–Whitney *U* test.

^#^p-values resulting from Kruskal–Wallis test.

p-values resulting from ANOVA.

Data of specimen measurements are reported as median (interquartile range).

**Fig. 2:**
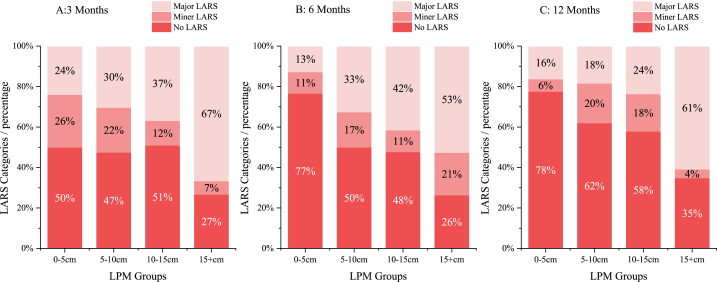
Distribution of LARS categories in different LPM groups.

#### 3.1.2 Multivariate analysis

To maximise the utilisation of the repeated measurements, we conducted a multivariate analysis with generalised estimation equations. LARS scores obtained during the three follow-ups were used as the dependent variable. Based on the LPM median value of 9.5 cm, LPM data were divided into two categories, viz. <10 and ≥ 10 cm), with tumour location (with 8 cm as the dividing point), radiotherapy, and follow-up time as the predictive factors and BMI as the covariate (Table 3). All the predictive factors included in the model were statistically significant. LPM <10 cm (odds ratio [OR] = 0.035, p = 0.006) and no radiotherapy (OR = 0.002, p = 0.001) were identified as protective factors for LARS score, while tumour location ≤8 cm (OR = 1.46, p < 0.001), postoperative time of 3 months (OR = 3.20, p < 0.001), and postoperative time of 6 months (OR = 1.703, p < 0.001) were identified as risk factors for LARS score.

**Table 3:** Multivariate risk factor analysis for LARS score adjusted for types of LPM using generalised estimating equation.

Variables	Full analysis (n = 376)		Radiotherapy history (n = 51)		Tumour position ≤ 8 cm (n = 200)	
	Odds ratio (CI)	p-value	Odds ratio (CI)	p-value	Odds ratio (CI)	p-value
**LPM**						
< **10 cm**	0.035 (0.003–0.379)	0.006	85.379 (0.004–1810018.27)	0.382	0.046 (0.002–1.101)	0.057
≥ **10 cm**	1		1		1	
**Radiotherapy**						
**No**	0.002 (<0.001–0.072)	0.001	–		0.005 (<0.001–0.244)	0.007
**Yes**	1				1	
**Tumour position (cm)**						
**≤ 8 cm**	142.656 (12.312–1652.886)	<0.001	0.068 (0.001–132.114)	0.487	–	
> **8 cm**	1		1			
**Follow-up**						
**3 months**	320.809 (43.37–2373.06)	<0.001	259.009 (0.023–2920223.717)	0.243	36.620 (1.664–805.989)	0.022
**6 months**	71.228 (13.401–378.594)	<0.001	0.538 (0.001–263.133)	0.844	30.093 (2.856–317.120)	0.007
**12 months**	1		1		1	
**BMI**	1.581 (1.096–2.279)	0.014	5.735 (1.013–32.453)	0.048	2.682 (1.565–4.598)	<0.001

Radiotherapy history and low tumour location have been recognised as important risk factors for LARS. Therefore, we conducted a subgroup analysis among the two patient groups. The results of the generalised estimation equation model showed that the protective effect of LPM <10 cm was not statistically significant in subgroup analysis. In the subgroup of tumour location ≤8 cm, the OR was 0.046, p = 0.057. Meanwhile, radiotherapy (OR = 0.005, p = 0.007), BMI (OR = 5.735, p = 0.048) and postoperative time (3 months: OR = 3.76, p < 0.001; 6 months: OR = 2.96, p < 0.001) were still significant. Among radiotherapy patients, the OR was 85.379 (p = 0.382), but BMI (OR = 5.735, p = 0.048) was still significant.

#### 3.1.3 Oncology compromise

**Fig. 3:**
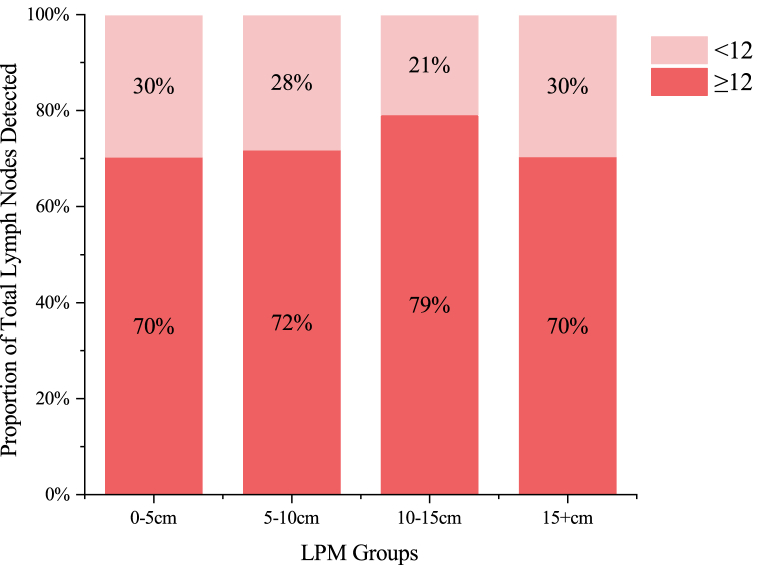
Total number of lymph nodes detected in different LPM groups.

As there was no tumour residue at the proximal margin of all specimens, we analysed the number of dissected lymph nodes to determine whether a shorter LPM would cause an oncology compromise. The total number of lymph nodes detected was 15.5 ± 6.4 in the <10-cm group and 15.1 ± 6.7 in the ≥10-cm group (p = 0.654). Considering that ≥12 is the internationally recognised standard for the number of lymph nodes detected for radical surgery, the proportion of lymph nodes detected at <12 in each LPM group in this study is basically consistent (p = 0.505) (Fig. 3).

## 4 Discussion

In this multi-centre, prospective, observational cohort study, both the subjective questionnaire and LARS score suggested that prolonged LPM would have a negative impact on postoperative bowel function, which persisted for 1 year after surgery. The major LARS risk factors confirmed in this study, such as radiotherapy and low tumour position, were consistent with recently reported findings [22,23,27]. To our knowledge, our study is the first to reveal and preliminarily verify that LPM, as an important indicator reflecting bowel volume loss, is associated with postoperative bowel function. Our findings suggest that the proximal margin of bowel resection during TME should be identified from the perspective of functional protection.

Shafik et al. demonstrated through a series of studies that the sigmoid colon is the major stool storage organ, and the rectosigmoid junction prevents faecal discharge into the rectum, which is often completely removed during TME surgery [28,29]. The removal of partial sigmoid and rectum by TME surgery will lead to the following functional changes: 1, decreased sigmoid volume can cause frequent bowel movement; 2, decreased sigmoid colon volume affects water absorption, resulting in looser faeces and increased possibility of incontinence; and 3, faeces might more easily enter the rectum and induce defecation reflex due to the loss of the rectosigmoid junction [30]. The LPM is a reliable indicator of sigmoid volume loss, and the phenomenon observed in this study is consistent with the abovementioned theory.

Both LDM and LPM exhibited completely different distribution trends. Owing to the dual limitations of the TME principle and anastomosis, LDM was primarily concentrated in the range of 2–4 cm, and LPM, without clinical guidelines, has a wide distribution. The surgeons in this study probably arbitrarily decided on the site of resecting the proximal bowel. This may be due to the lack of a length requirement for proximal margins in the classic TME principles and other guidelines and for either oncological or surgical safety reasons. Although retaining more sigmoid colon is beneficial for postoperative bowel function, it is also necessary to consider oncological and surgical safety in determining the site of proximal and distal bowel resection during TME. Unlike for the LDM, we did not find a definite specification for LPM in the relevant literature, guidelines or expert consensus published in Chinese and English.

As significant individual and racial differences exist in sigmoid length, the residual sigmoid length should be superior to LPM as a predictor, and even with the same LPM, patients with a shorter sigmoid are more susceptible. Madiba et al. confirmed through autopsy that the sigmoid colon of African and Indian vegetarian populations was significantly longer than that of White people [31]. In this study, the length of the sigmoid colon was 22.6 ± 9.2 cm in White individuals and 29.6 ± 12.1 cm in Black individuals, whereas Ran et al. reported that the length of the sigmoid colon in Chinese people was much longer, reaching 35.5 ± 12.1 cm [32]. When we analysed the proportion of patients with major LARS in the literature published in English in recent years, we found an interesting trend. Most studies from Europe reported a proportion of >50%, whereas all studies from Asia reported a proportion of <50%, except studies primarily on preoperative radiotherapy (appendix 4). Our theory may partially explain this difference in postoperative bowel function between Asia and Europe, i.e. patients from Asia with a more vegetarian diet have a longer residual sigmoid colon.

Although the functional protective effect of LPM discovered in our subgroup analysis was not as significant as that of radiotherapy and low tumour location, LPM remains an important factor for functional protection. Compared with traditional LARS risk factors such as obesity, radiotherapy and low tumour position, the surgeon can proactively improve postoperative bowel function by controlling LPM. Moreover, subgroup analysis of LARS revealed a significant difference in people with tumour location ≤8 cm (p = 0.057). Based on our data, we recommend implementing steps to preserve the maximum possible length of the sigmoid colon during APS. To achieve radical resection of the tumour and lymph node dissection, it is recommended to control LPM within the range of 10 cm. If an additional portion of the sigmoid colon needs to be excised due to the presence of intestinal ischaemia, the LPM should not exceed 15 cm. If a large amount or the entire sigmoid colon needs to be removed, the patient should be made aware of the significant possibility of postoperative bowel dysfunction, and close follow-up and active functional therapy must be conducted for such patients.

Regarding the safety of the 10 cm length, it is known that the site of the cut-off margin should be determined according to the principles of oncological and surgical safety in APS. Although oncology follow-up results were lacking in this study, our data also suggest that limiting the proximal margin to 10 cm causes limited oncology compromise.

### 4.1 Study limitations

As discussed earlier, the volume of the residual colon may have an even greater impact on postoperative bowel function. Therefore, it is essential to measure the length of the residual bowel, such as the length from the descending junction to the anastomosis site. However, intraoperative measurement of bowel remnants is neither easy in laparoscopic surgery nor a standard practice during APS. Moreover, further study is needed for the intraoperative identification of the origin of the sigmoid colon [29,33]. Therefore, we did not attempt to measure this variable. Hence, further studies with a standard measurement protocol of the residual colon are required to test our hypothesis.

Only 13.6% (51/376) of the patients in this study received radiotherapy, leading to a reduction in the statistical power of subgroup analysis for radiotherapy patients.

Our findings may provide some evidence of proximal margin confirmation for the future surgical practice of rectal cancer. Nevertheless, to validate this finding, well-designed randomised controlled trials are required, and our research team has designed and registered the Bas-1904 study on the ClinicalTrials.gov website to further validate the findings of this study.

## 5 Conclusion

In APS for rectal cancer, bowel volume loss is an important factor resulting in postoperative bowel dysfunction. Controlling the length of the proximal incision margin to <10 cm may help improve postoperative bowel function.

## Author contribution statement

FAN Liu: Conceived and designed the experiments; Performed the experiments; Analysed and interpreted the data; Contributed reagents, materials, analysis tools or data; Wrote the paper.

Peng Guo; Quan Wang; Fujun Chen; Wenyong Wu; Xiangqian Su; Guiying Wang; Zhouman Yu; Jianlong Jiang; Feng Liang; Dechang Diao; Zhikang Chen; Yuanting Liu; Fanqiang Meng; Ning Ning: Conceived and designed the experiments; Performed the experiments; Contributed reagents, materials, analysis tools or data.

Yingjiang Ye: Conceived and designed the experiments.

## Data availability statement

Data associated with this study has been deposited at ClinicalTrials.gov under the accession number NCT03009747.

## Additional information

Supplementary content related to this article has been published online at [URL].

## Declaration of competing interest

The authors declare that they have no known competing financial interests or personal relationships that could have appeared to influence the work reported in this paper.

## Figures and Tables

**figs1:**
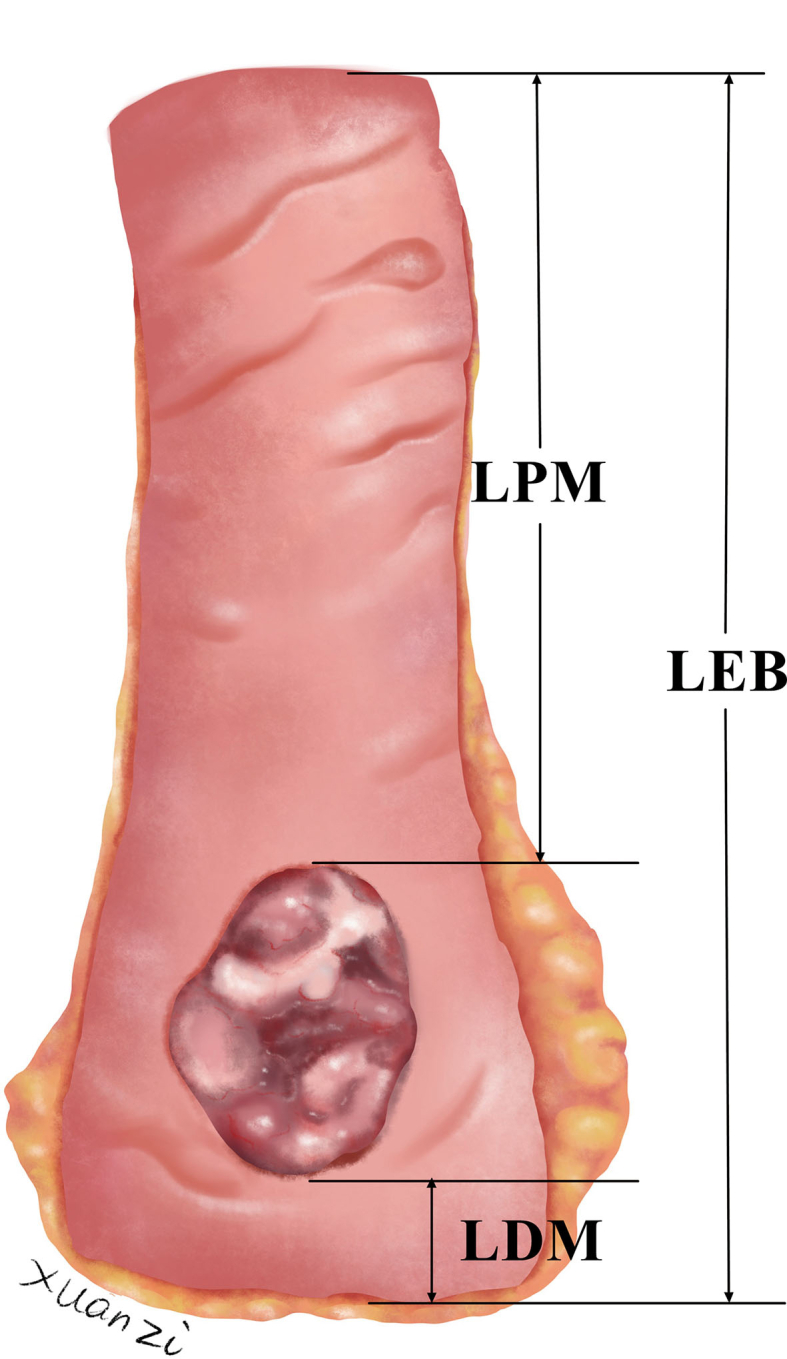


**figs2:**
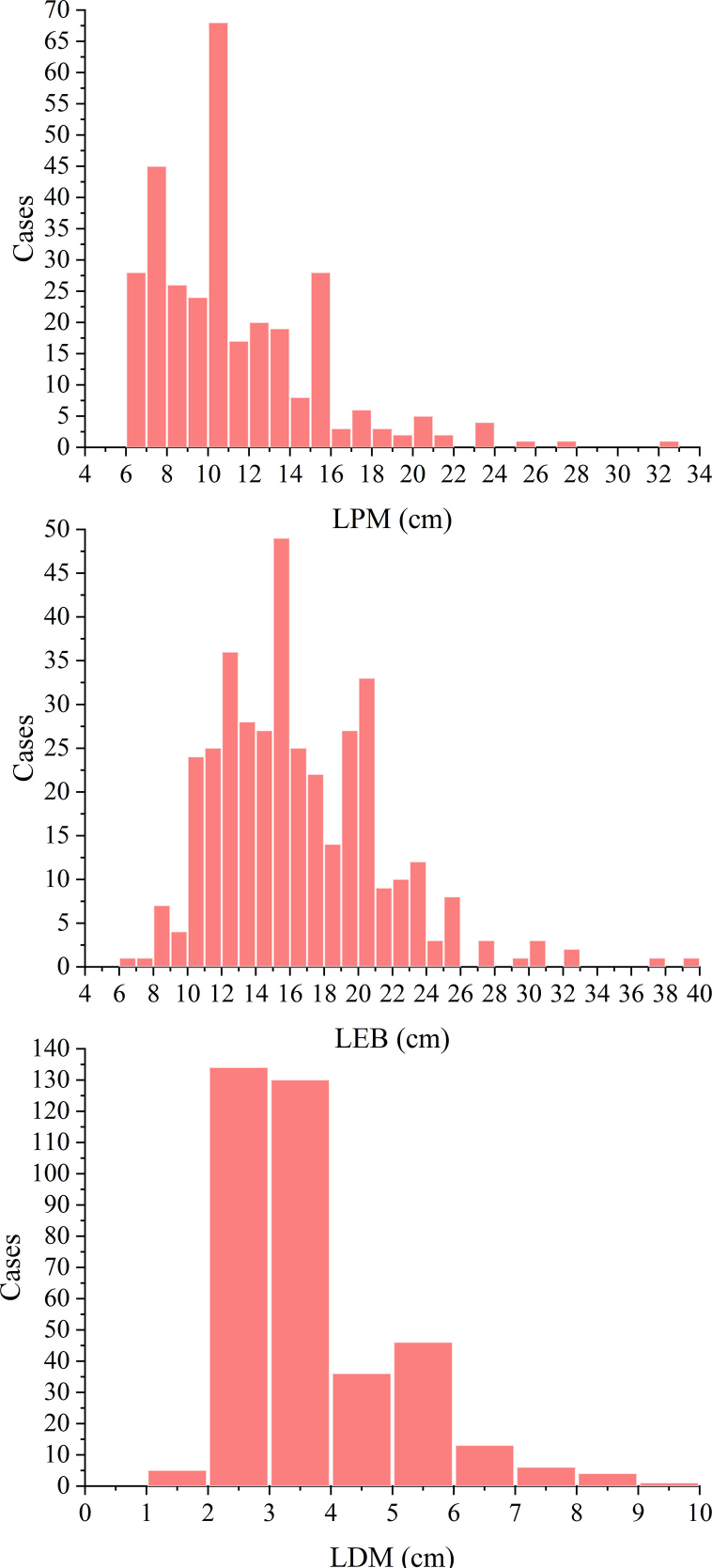


## Appendix 1 Schematic of specimen measurement

See Appendix 1.tif.

LDM: length from the distal margin of the specimen to the distal margin of the tumour.

LPM: length from the proximal margin of the specimen to the proximal margin of the tumour.

LEB: the overall length of the excised bowel.

## Footnotes

Appendix A Supplementary data to this article can be found online at https://doi.org/10.1016/j.heliyon.2023.e17630.
