# Supplementary material for: Excessive bowel volume loss during anus-preserving surgery for rectal cancer affects the bowel function after operation: A prospective observational cohort study (Bas-1611)
Source: Heliyon. 2023 Jul 6;9(7):e17630. doi: 10.1016/j.heliyon.2023.e17630 (PMC10362271; doi:10.1016/j.heliyon.2023.e17630)
Supplement: Multimedia component 1 [file mmc1.docx]

Appendix 1 Schematic of specimen measurement

| See Appendix 1.tif |
| --- |

LDM: length from the distal margin of the specimen to the distal margin of the tumour.

LPM: length from the proximal margin of the specimen to the proximal margin of the tumour

LEB: the overall length of the excised bowel

Appendix 2 Length distribution of specimen measurement variables

| See Appendix 2.tif |
| --- |

LDM: length from the distal margin of the specimen to the distal margin of the tumour.

LPM: length from the proximal margin of the specimen to the proximal margin of the tumour

LEB: the overall length of the excised bowel

| **Variables** | Do you have abnormal bowel movements? | | | Are you satisfied with your current defecation status? | | | Does your defecation status affect your quality of life? | | |
| --- | --- | --- | --- | --- | --- | --- | --- | --- | --- |
| **3 Months (n=257)** | Yes  n=177 | No  n=80 | P value | Yes  n=167 | No  n=90 | P value | Yes  n=108 | No  n=149 | P value |
| **LDM (cm)** | 3.0 (1.0-10.0) | 3.0 (1.0-8.0) | 0.535 | 3.0 (1.0-10.0) | 3.0 (1.0-8.0) | 0.456 | 3.0 (2.0-10.0) | 3.0 (1.0-8.0) | 0.722 |
| **LPM (cm)** | 9.0 (2.0-32.0) | 8.5 (3.0-23.0) | 0.635 | 9.0 (2.0-32.0) | 9.0 (3.0-25.0) | 0.316 | 10.0 (2.0-25.0) | 8.0 (3-32.0) | 0.078 |
| **LEB (cm)** | 15.0 (6.0-37.0) | 15.0 (9.0-29.0) | 0.877 | 15.0 (8.0-37.0) | 15.0 (6.0-32.0) | 0.722 | 15.0 (6.0-32.0) | 15.0 (7.0-37.0) | 0.152 |
| **6 Months (n=275)** | Yes  n=190 | No  n=85 | P value | Yes  n=185 | No  n=90 | P value | Yes  n=108 | No  n=167 | P value |
| **LDM (cm)** | 3.0 (1.0-8.0) | 3.0 (2.0-10.0) | 0.011 | 3.0 (1.0-8.0) | 3.0 (2.0-10.0) | 0.303 | 3.0 (2.0-10.0) | 3.0 (1.0-8.0) | 0.118 |
| **LPM (cm)** | 10.0 (2.0-23.0) | 8.0 (2.0-32.0) | 0.004 | 9.0 (2.0-32.0) | 10.0 (3.0-21.0) | 0.001 | 10.0 (3.0-23.0) | 8.0 (2.0-32.0) | < 0.001 |
| **LEB (cm)** | 15.0 (6.0-32.0) | 15.0(9.0-39.0) | 0.429 | 15.0 (6.0-39.0) | 15.0 (8.0-32.0) | 0.078 | 16.0 (8.0-30.0) | 15.0 (6.0-39.0) | 0.028 |
| **12 Months (n=311)** | Yes  n=194 | No  n=117 | P value | Yes  n=217 | No  n=94 | P value | Yes  n=209 | No  n=102 | P value |
| **LDM (cm)** | 3.0 (1.0-10.0) | 3.0 (2.0-7.0) | 0.366 | 3.0 (1.0-10.0) | 3.0 (1.0-9.0) | 0.149 | 3.0 (1.0-10.0) | 3.0 (1.0-9.0) | 0.249 |
| **LPM (cm)** | 10.0 (2.0-32.0) | 8.0 (2.0-23.0) | 0.011 | 9.0 (2.0-25.0) | 10.0 (2.0-32.0) | 0.060 | 10.0 (2.0-32.0) | 9.0 (2.0-25.0) | 0.035 |
| **LEB (cm)** | 15.0 (6.0-37.0) | 15.0 (8.0-27.0) | 0.139 | 15.0 (6.0-32.0) | 15.0 (8.0-37.0) | 0.202 | 15.0 (8.0-37.0) | 15.0 (6.0-32.0) | 0.141 |
| LDM: length of the distal margin, LPM: length of the proximal margin, LEB: length of excised bowel  Data were reported as median (interquartile range)  The P value comes from the Mann–Whitney Test. | | | | | | | | | |

Appendix 3. Follow-up results of three subjective questionnaires

| Appendix 4. The proportion of major LARS reported in recent literatures | | | | | |
| --- | --- | --- | --- | --- | --- |
| Author | Nation | Sample Size (N) | Median follow-up (Months) | Ratio of neoadjuvant radiotherapy (%) | Ratio of Major LARS (%) |
| European | | | | | |
| Bohlok, et al.^1^ | Belgium | 43 | 71 | 90.1 | 72.1 |
| Keane, et al. ^2^ | New Zealand | 186 | 52 | 44 | 52 |
| van Heinsbergen, et al. ^3^ | Netherlands | 350 | 60 | 72 | 55.4 |
| Keane, et al. ^4^ | New Zealand  Sweden  Denmark | 82 | 50 | 25.6 | 66 |
| Samalavicius et al.^5^ | Lithuania | 108 | 12 | 49 | 56 |
| Asain | | | | | |
| Fan, et al. ^6^ | China | 100 | 23 | 14 | 43 |
| Tan, et al. ^7^ | China | 38 | 3 | 60.5 | 45.5 |
| Ekkarat, et al. ^8^ | Thailand | 129 | 36 | 38 | 17.8 |
| Beppu, et al. ^9^ | Japan | 87 | 78 | 100 | 51.4 |
| Liang, et al. ^10^ | Malaysia | 76 | 34.8 | 30.3 | 32.9 |
| This study (data from 12 months) | China | 311 | 12 | 14.5 | 22.5 |

1. Bohlok, A., et al., *The burden of low anterior resection syndrome on quality of life in patients with mid or low rectal cancer.* Support Care Cancer, 2019.

2. Keane, C., et al., *Comparison of bowel dysfunction between colorectal cancer survivors and a non-operative non-cancer control group.* Colorectal Dis, 2020.

3. van Heinsbergen, M., et al., *Functional bowel complaints and quality of life after surgery for colon cancer: prevalence and predictive factors.* Colorectal Dis, 2019. **22**(2): p. 136-145.

4. Keane, C., et al., *Functional outcomes from a randomized trial of early closure of temporary ileostomy after rectal excision for cancer.* Br J Surg, 2019. **106**(5): p. 645-652.

5. Samalavicius, N.E., et al., *Validity and reliability of a Lithuanian version of low anterior resection syndrome score.* Tech Coloproctol, 2016. **20**(4): p. 215-220.

6. Liu, F., et al., *Risk factor analysis of low anterior resection syndrome after anal sphincter preserving surgery for rectal carcinoma.* Chinese Journal of Gastrointestinal Surgery, 2017. **20**(3): p. 289-294.

7. Tan, S.H., et al., *Exploring bowel dysfunction of patients following colorectal surgery: A cohort study.* J Clin Nurs, 2018.

8. Ekkarat, P., et al., *Factors determining low anterior resection syndrome after rectal cancer resection: A study in Thai patients.* Asian J Surg, 2016. **39**(4): p. 225-31.

9. Beppu, N., et al., *Long-Term Functional Outcomes of Total Mesorectal Excision Following Chemoradiotherapy for Lower Rectal Cancer: Stapled Anastomosis versus Intersphincteric Resection.* Dig Surg, 2016. **33**(1): p. 33-42.

10. Liang, L.S., et al., *Risk factors associated with low anterior resection syndrome: a cross-sectional study.* Ann Coloproctol, 2022.
