# Supplementary material for: Excessive bowel volume loss during anus-preserving surgery for rectal cancer affects the bowel function after operation: A prospective observational cohort study (Bas-1611)
Source: Heliyon. 2023 Jul 6;9(7):e17630. doi: 10.1016/j.heliyon.2023.e17630 (PMC10362271; doi:10.1016/j.heliyon.2023.e17630)
Supplement: Multimedia component 2 [file mmc2.pdf]

The LARS Score - Scoring Instructions

Add the scores from each 5 questions to one final score.

Do you ever have occasions when you cannot control your flatus (wind)?

- ☐ No, never **0**
- ☐ Yes, less than once per week **4**
- ☐ Yes, at least once per week **7**

Do you ever have any accidental leakage of liquid stool?

- ☐ No, never **0**
- ☐ Yes, less than once per week **3**
- ☐ Yes, at least once per week **3**

How often do you open your bowels?

- ☐ More than 7 times per day (24 hours) **4**
- ☐ 4-7 times per day (24 hours) **2**
- ☐ 1-3 times per day (24 hours) **0**
- ☐ Less than once per day (24 hours) **5**

Do you ever have to open your bowels again within one hour of the last bowel opening?

- ☐ No, never **0**
- ☐ Yes, less than once per week **9**
- ☐ Yes, at least once per week **11**

Do you ever have such a strong urge to open your bowels that you have to rush to the toilet?

- ☐ No, never **0**
- ☐ Yes, less than once per week **11**
- ☐ Yes, at least once per week **16**

Total Score: ______

Interpretation:

0-20: **No LARS**
21-29: **Minor LARS**
30-42: **Major LARS**
